# Magnesium-Calcium Exchange-Driven Elastic Properties of Alkali Charge-Balanced Aluminosilicate-Graphene Nanocomposites

**DOI:** 10.3390/nano16120778

**Published:** 2026-06-19

**Authors:** Mohammadreza Izadifar, Peter Thissen, Osama Ahmed Mohamed, Neven Ukrainczyk, Mohammadjavad Boroumandi, Moaz Omar, Anas Omar, Eduardus Koenders

**Affiliations:** 1 Institute of Construction and Building Materials, Technical University of Darmstadt, Franziska-Braun-Str. 3, 64287 Darmstadt, Germany; ukrainczyk@wib.tu-darmstadt.de (N.U.); koenders@wib.tu-darmstadt.de (E.K.); 2 Institute of Concrete Structures and Building Materials, 76131 Karlsruhe, Germany; peter.thissen@kit.edu; 3 Department of Architecture/Department of Civil Engineering, College of Engineering, Abu Dhabi University, Abu Dhabi P.O. Box 59911, United Arab Emirates; osama.mohamed@adu.ac.ae

**Keywords:** alkali charge-balanced C–A–S–H, partial decalcification, Mg^2+^ substitution, reduced graphene oxide (rGO), density functional theory, elastic properties, cementitious nanocomposites

## Abstract

Magnesium–rich environments are frequently encountered in cementitious systems, including the use of high–Mg raw materials in clinker production, cement–clay interfaces relevant to nuclear waste disposal, and exposure of cement–based materials to seawater, where progressive decalcification can substantially alter the structure and durability of calcium aluminosilicate hydrate (C–A–S–H) phases. In this study, density functional theory (DFT) calculations were employed to investigate the combined effects of interlayer and intralayer partial decalcification, Mg^2+^ substitution, and reinforcement with epoxy– and hydroxyl–functionalized reduced graphene oxide (rGO) on the structural stability and elastic properties of alkali charge–balanced C–A–S–H under dry and hydrated conditions. Adsorption–energy calculations reveal thermodynamically favorable interactions between functionalized rGO and silicate hydrate species in the presence of Mg^2+^, with hydroxyl/rGO promoting stronger interfacial stabilization and epoxy/rGO preserving greater graphene lattice integrity. The results demonstrate that Mg^2+^ substitution together with rGO intercalation generally enhances the mechanical response of partially decalcified structures through structural densification and interfacial cohesion. Relative to dry systems, hydration further improves elastic performance, increasing Young’s modulus and bulk modulus by 1–11% and 4–19%, respectively, for interlayer decalcified nanocomposites, while intralayer configurations exhibit stronger but model–dependent enhancements of up to ≈22% and ≈33%. Compared with untreated systems, rGO–treated nan–composites exhibit enhanced stiffness, with Young’s modulus and bulk modulus increasing by up to ≈22% and ≈15%, respectively. Overall, these findings provide atomistic insights into stabilization mechanisms in partially decalcified alkali charge–balanced C–A–S–H systems and identify Mg^2+^–rGO incorporation as a promising strategy for mitigating decalcification–induced degradation in durable low–carbon cementitious nanocomposites.

## 1. Introduction

Concrete remains the most widely used construction material globally [1]; however, its production is a major contributor to anthropogenic CO_2_ emissions. The primary source of these emissions arises from clinker production, during which limestone calcination releases substantial amounts of carbon dioxide [2]. Global CO_2_ emissions from cement production are now estimated at approximately 4.10 Gt per year, compared to 2.22 Gt per year a decade ago [1], reflecting a rapid and concerning increase in the sector’s carbon footprint. With increasing environmental awareness, the development of alternative binders and sustainable nanocomposite materials has become one of the most active research directions in cement science. The central focus of this effort lies in understanding and improving the nanostructure behavior of calcium silicate hydrates (C–S–H), the principal binding phase in hydrated Portland cement [3]. The C-S-H gel is considered the key component of concrete, accounting for 60–70% of fully hydrated cement paste. It is ultimately responsible for the mechanical behavior of cementitious materials. C-S-H gels have calcium-to-silicate ratios ranging from 0.7 to 2.3 [4].

C–S–H determines the strength, stiffness, and long–term durability of cementitious materials and has therefore been extensively studied as the backbone of concrete microstructure [3]. The atomic structure of C–S–H is typically modeled on naturally occurring tobermorite minerals, especially the 14Å polymorph [5], whose layered silicate–calcium framework represents the fundamental building unit of the gel [6]. Despite its structural relevance, C–S–H suffers from limited chemical stability under decalcifying or aggressive environmental conditions [7]. When exposed to leaching or sulfate attack, calcium ions are progressively released from the interlayer, causing the Ca/Si ratio to decrease [8,9]. This transformation induces shortening of the silicate chains, a higher degree of polymerization, and the formation of an amorphous, silica–rich phase. Liu et al. [10] showed that this decalcification primarily initiates in the interlayer region, where the weakly bound calcium ions are most susceptible to dissolution [11,12,13,14], while the intralayer calcium sites remain comparatively stable. As a result, the mechanical stiffness decreases substantially, particularly along the c–axis of the layered structure. The loss of calcium thus leads to weakening of the interlayer cohesion and a pronounced reduction in the elastic moduli. This limitation highlights the importance of magnesium substitution, as Mg^2+^, with its smaller ionic radius and higher charge density, forms shorter and stronger metal–oxygen bonds that densify the silicate framework and enhance structural cohesion. Together, these studies indicate that decalcification and insufficient charge balancing significantly weaken C–S–H, and that magnesium doping offers a promising route to restore stability and maintain mechanical integrity under chemically aggressive conditions. Santos Rego et al. [15] also used a DFT computational approach to evaluate the substitution of magnesium at various calcium positions (interlayer and intralayer) in tobermorite 9Å (dry) and 11Å (hydrated) [16,17], showing that Mg incorporation in the interlayer significantly improves cohesive strength and enhances the elastic properties. The substitution in interlayer sites led to a notable increase in bulk modulus and shear modulus and to shorter Mg–O bonds that promote structural compaction. In contrast, intralayer substitution induced pronounced anisotropy, which could adversely affect the mechanical durability. Li et al. [18] reported that the density and stiffness of tobermorite 11Å are strongly controlled by the Ca/Si ratio, and that removing calcium without compensation makes the lattice porous and unstable. They further demonstrated that interlayer magnesium not only enhances the chemical stability of the decalcified framework but also optimizes the electronic configuration of the silicate chains, making magnesium substitution an effective way to counteract decalcification and improve the stiffness and durability of C–S–H.

Recent studies aim to enhance concrete’s mechanical performance by developing cement–based nanocomposites. Incorporating graphene [19,20,21,22,23,24], particularly reduced graphene oxide (rGO) [25,26], has proven promising due to graphene‘s exceptional mechanical properties [27,28,29]. Izadifar et al. [4,6] pioneered the use of first-principles modeling to explore the interaction mechanism of rGO functionalized with cementitious C–S–H gel moieties, revealing that such functionalized rGO plays a critical role in enhancing elastic properties. Parallel to these nanocomposite–driven advancements, promising studies have been conducted on synthesizing magnesium–silicate–hydrate (M–S–H). Bernad et al. [30] investigated the role of magnesium in C–S–H stability through batch experiments in which magnesium oxide or magnesium chloride was introduced into C–S–H with a Ca/Si ratio of 0.8. In a subsequent experimental study, Bernad et al. [31] investigated the possible incorporation of calcium into M–S–H and of magnesium into C–S–H. In their batch experiments, M–S–H was synthesized in calcium–containing solutions, while C–S–H was synthesized in the presence of magnesium. These experimental studies clarified the potential for ion exchange between the phases and provide a basis for the present work, which advances this understanding by examining Mg incorporation in C–S–H using atomistic modeling.

Recently, the mechanisms governing the interactions between functionalized rGO and cementitious C–S–H gel moieties during the fabrication of cementitious nanocomposite materials, and their consequent effects on elastic properties, have been reported [4,6,32]. Puertas et al. [33,34] reported a model for alkali charge-balanced C–A–S–H gels derived from slag. In parallel, Vespa et al. [35] carried out a detailed structural characterization of magnesium (sodium) aluminum silicate hydrate (M–(N)–A–S–H) phases using X–ray absorption near–edge spectroscopy, providing important insights into their local coordination environments and structural features. However, a comprehensive atomistic understanding of these interactions, particularly in partially decalcified and alkali charge–balanced Al–substituted C–S–H systems, as well as their influence on structural stability and elastic properties, remains largely unexplored. Building on these developments, this study employs density functional theory (DFT) calculations to first quantify the adsorption energies and interaction mechanisms between hydroxyl– and epoxy–functionalized reduced graphene oxide (rGO) lattices and decalcified C–S–H systems. Subsequently, the combined effects of interlayer or intralayer decalcification, together with the intercalation of interstitial rGO, on the structural stability and mechanical properties of alkali charge-balanced Al-substituted C–S–H are systematically investigated. The systems were modeled in hydrated and dry (dehydrated) states using tobermorite 14Å, representative of a C–S–H system. The dry model was obtained by removing interlayer water, yielding a reduced basal spacing comparable to tobermorite 9Å [36,37]. These phases are widely accepted structural analogs for the calcium– (sodium– or potassium–) aluminosilicate–hydrate gels [38] formed in alkali-activated slag (AAS) systems [39], namely [M–C–(K–)A–S–H] and [M–C–(Na–)A–S–H]. Following the tobermorite-based structural description of C–(Na– or K–)A–S–H gels proposed by Myers et al. [40], atomic models were constructed by introducing controlled decalcification within the tobermorite framework to reproduce Ca-deficient environments typical of AAS binders. More recently, Izadifar et al. [32] employed DFT calculations to investigate the interfacial bonding mechanisms and elastic properties of alkali charge-balanced C–(Na–)A–S–H and C–(K–)A–S–H nanocomposites reinforced with hydroxyl- and epoxy–functionalized rGO lattices. The results demonstrated that functionalized rGO can significantly enhance the structural stability and mechanical performance of AAS–derived binding phases. Building upon experimental observations of Mg^2+^ incorporation in structural characterization of magnesium (sodium) aluminum silicate hydrate (M–(N)–A–S–H) phases reported by Vespa et al. [35], partial Mg^2+^ substitution at Ca^2+^ sites was subsequently implemented to evaluate the influence of Mg–modified C–(Na– or K–)A–S–H structures in the presence of rGO lattice. Overall, this work aims to clarify how decalcification and Mg^2+^ substitution [41], together with rGO lattices, influence the structural stability and mechanical behavior of alkali–activated systems, thereby contributing to the development of next–generation low-carbon concrete materials with enhanced mechanical performance.

## 2. Methodology and Computational Models

### 2.1. Computational Details and Structural Preparation

The computational framework followed established procedures for modeling reinforced C–S–H nanocomposites, integrating full structural relaxation with adsorption–energy evaluations and elasticity property calculations. All simulations in this study were performed using density functional theory (DFT) [42] via the Vienna Ab initio Simulation package (VASP, version 6.4.1) [43,44,45,46,47,48,49], employing the projector–augmented–wave (PAW) method to describe electron–ion interactions [50]. Exchange–correlation effects were treated using the generalized–gradient approximation (GGA) parameterized by Perdew, Burke, and Ernzerhof (PBE) [51]. A plane–wave cutoff of 400 eV ensured energy convergence, while electronic self–consistent cycles were converged to 10^−8^ eV. Ionic relaxation was continued until the maximum residual forces on all atoms were below 10^−6^ eV Å^−1^, allowing simultaneous optimization of lattice vectors and atomic coordinates. The Brillouin zone was sampled using a 2 × 2 × 1 Monkhorst–Pack grid [52], and all structural analyses and visualizations were performed using VESTA [53].

Reference structures representing hydrated and dry C–S–H systems were used as base models [36,37]. The hydrated reference structure was based on crystalline tobermorite 14Å (plombierite, Ca_5_Si6O_16_(OH)_2_·7H_2_O; Ca/Si = 0.83), which is widely accepted as a structural analog of C–S–H. The structure consists of calcium–oxygen layers, dreierkette silicate chains, and an interlayer region containing water molecules and charge–balancing cations. The initial model was adopted from the experimentally refined B11b structure with lattice parameters of a = 6.735Å, b = 7.425Å, c = 27.987Å, α = 90°, β = 90°, and γ = 123.25°. Dry models were generated by removing interlayer water molecules from the hydrated structure, resulting in a basal spacing comparable to tobermorite 9Å. Subsequently, partial interlayer or intralayer decalcification was introduced by selectively removing Ca^2+^ ions, followed by Mg^2+^ substitution and alkali charge balancing using Na^+^ or K^+^. A series of model configurations (models 1–12) were generated to investigate the effects of interlayer or intralayer decalcification, Mg^2+^ substitution, as well as the influence of rGO reinforcement in alkali–hydrated and dry C–A–S–H systems. In the present models, partial decalcification was introduced through the removal of Ca^2+^ ions from either the interlayer or intralayer region of the reference tobermorite structure, followed by Mg^2+^ substitution. The resulting Mg^2+^ substitution levels correspond to approximately 27% for the interlayer-decalcified nanocomposite models and 72% for the intralayer–decalcified nanocomposite models. For the untreated alkali C–A–S–H models, the corresponding Mg^2+^ substitution levels are approximately 20% and 80% for the interlayer– and intralayer–decalcified models, respectively. These models include interlayer or intralayer Mg–doping variants and hydroxyl– and epoxy–functionalized rGO lattices. The rGO nanosheets were positioned parallel to the silicate surface to represent realistic interfacial interactions. The hydroxyl/rGO and epoxy/rGO models correspond to a functionalization density of approximately 11.1%. This level of functionalization is representative of partially reduced graphene oxide and provides chemically active interfacial sites while preserving the structural integrity of the graphene lattice [54,55]. Charge neutrality of the system was maintained through the symmetrical distribution of interlayer Na^+^ and K^+^. Additionally, interlayer free hydroxyl groups were introduced to balance the charge between Mg^2+^ or Ca^2+^ and the dangling oxygen atoms of the silicate tetrahedra chains.

### 2.2. Computation of Adsorption Energy

The adsorption energy (*E*_ad_) [6,32] of C–S–H gel on the rGO substrate was computed from the total DFT energies according to

$$E_{\mathrm{ads}}=E_{\mathrm{tot}}(\mathrm{composite})-[E_{\mathrm{tot}}(C–S–H\;\mathrm{gel})+E_{\mathrm{tot}}(\mathrm{rGO}–\mathrm{substrate})]$$

where *E*ₜₒₜ (composite), *E*ₜₒₜ (C–S–H gel), and *E*ₜₒₜ (rGO–substrate) represent the total energies of the relaxed composite, isolated gel, and rGO nanosheet, respectively. A negative *E*_ads_ value indicates thermodynamically favorable adhesion. Long-range dispersion interactions were incorporated using Grimme’s DFT-D3 [56,57] correction to accurately describe van der Waals contributions between the rGO nanosheet and the hydrated gel.

### 2.3. Computation of Elastic Constants

The elastic constants were calculated using a DFT computational approach. All configurations were first subjected to full structural relaxation to ensure accurate and reliable results. Then, lattice vector perturbations were applied to determine the resulting forces, from which the stiffness tensor C was evaluated. The compliance tensor S was subsequently obtained as its inverse, [*S*] = [*C*]^−1^. Within the Voigt–Reuss–Hill (VRH) approximation [58,59,60], the macroscopic elastic properties, namely Young’s modulus (E), Poisson’s ratio (ν), shear modulus (G), and bulk modulus (K), were derived from the computed elastic constants. The corresponding equations and computational procedures follow those reported in our previous studies and are therefore omitted here for brevity [4].

## 3. Results and Discussion

### 3.1. Hydroxyl/rGO with Silicate Hydrate Units in the Presence of Mg Ions

**Figure 1:**
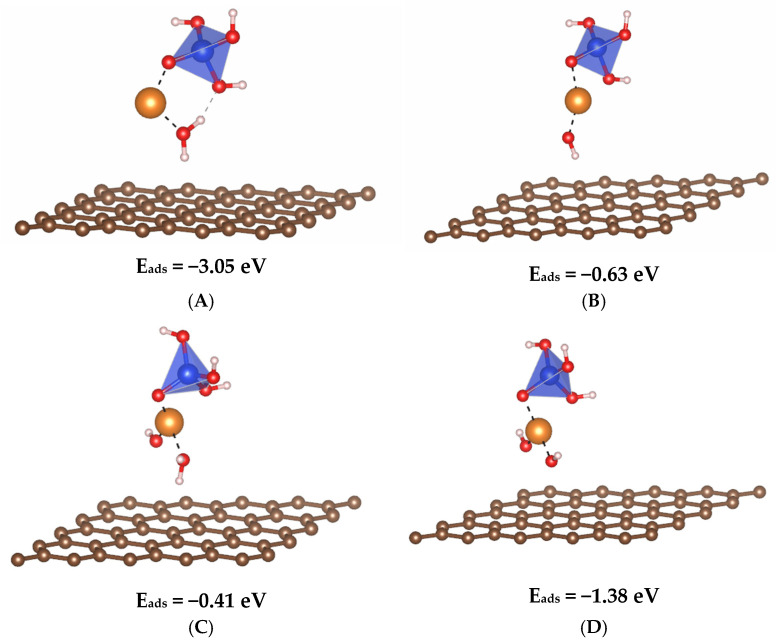
Optimized adsorption configurations of hydroxyl-functionalized rGO interacting with silicate species and Mg^2+^. (**A**) Mg^2+^ with Si(OH)_4_ adsorbed on the hydroxyl/rGO surface. (**B**) Mg^2+^ with SiO(OH)_3_ adsorbed on the hydroxyl/rGO surface. (**C**) Mg^2+^ with Si(OH)_4_ and an additional OH^−^ group adsorbed on the hydroxyl/rGO surface. (**D**) Mg^2+^ with SiO(OH)_3_ and an additional OH^−^ group adsorbed on the hydroxyl/rGO surface. Corresponding adsorption energies (E_ads_) calculated using DFT calculations are indicated for each configuration.

Figure 1 presents the optimized adsorption configurations of hydroxyl–functionalized rGO (hydroxyl/rGO) interacting with silicate hydrate species in the presence of Mg^2+^. Four representative configurations are considered, involving Si(OH)_4_ and SiO(OH)_3_ units, with and without additional hydroxyl groups.

In Figure 1A, the adsorption of Mg^2+^ coordinated with a neutral Si(OH)_4_ unit on the hydroxyl/rGO surface yields a highly stable configuration with an adsorption energy of −3.05 eV, indicating strong chemisorption. This high stability originates from a condensation mechanism at the hydroxyl/rGO interface, where a surface hydroxyl group dissociates and reacts with a proton from Si(OH)_4_ to form a water molecule. A similar process has been previously reported in Ca^2+^–containing system, where hydroxyl dissociation and proton transfer likewise led to water formation, contributing to interfacial stabilization and partial restoration of the graphene lattice [6]. Notably, for the same configuration, the adsorption energy in the presence of Ca^2+^ is significantly more negative than that of Mg^2+^, indicating stronger binding. This enhancement arises from the higher coordination capability and more effective charge transfer of Ca^2+^, which promotes stronger Coulomb interactions between the silicate unit and the rGO surface.

For the remaining configurations, a clear reduction in adsorption strength is observed compared to case (A). The interactions are characterized by hydroxyl dissociation from the rGO surface and the formation of an ionic bond between the dissociated hydroxyl and the dangling oxygen of the SiO(OH)_3_ unit, resulting in weaker interfacial bonding. Notably, hydroxyl dissociation from the rGO lattice remains energetically favorable even under charge–neutral conditions, as demonstrated by the configuration shown in Figure 1D. Upon introducing an additional hydroxyl group to the system, the adsorption energy further decreases to −0.41 eV for the Si(OH)_4_ system, while it increases moderately to −1.38 eV for the SiO(OH)_3_ configuration, reflecting the competing effects of charge redistribution from the rGO surface to the dissociated hydroxyl group near the lattice.

In comparison, the corresponding Ca^2+^-containing systems exhibit consistently more negative adsorption energies, indicating significantly stronger interactions. Overall, while Mg^2+^ contributes to interfacial stabilization, Ca^2+^ leads to markedly stronger adsorption across all comparable configurations.

### 3.2. Epoxy/rGO with Silicate Hydrate Units in the Presence of Mg Ions

**Figure 2:**
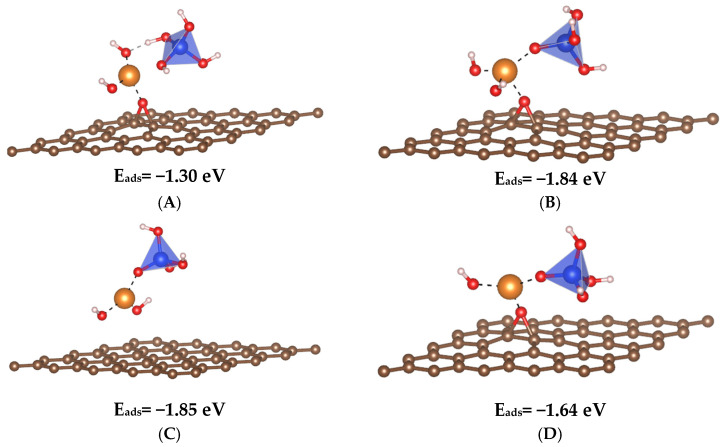
Optimized adsorption configurations of epoxy-functionalized rGO interacting with silicate species and Mg^2+^. (**A**) Mg^2+^ with Si(OH)_4_ and two additional OH^−^ groups adsorbed on the epoxy/rGO surface. (**B**) Mg^2+^ with SiO(OH)_3_ and two additional OH^−^ groups adsorbed on the epoxy/rGO surface. (**C**) Mg^2+^ with Si(OH)_4_ and an additional OH^−^ group adsorbed on the epoxy/rGO surface. (**D**) Mg^2+^ with SiO(OH)_3_ and an additional OH^−^ group adsorbed on the epoxy/rGO surface. Corresponding adsorption energies (E_ads_) calculated using DFT calculations are indicated for each configuration.

Figure 2 shows the optimized adsorption configurations of epoxy-functionalized rGO interacting with silicate hydrate units in the presence of Mg^2+^. Compared to hydroxyl/rGO, all configurations exhibit relatively similar adsorption energies, ranging from −1.30 to −1.84 eV, indicating moderate but consistent interfacial interactions. The configuration involving Si(OH)_4_ with two additional hydroxyl groups (charge–neutral condition) shows the lowest stability (−1.30 eV), whereas the configurations associated with SiO(OH)_3_ and one hydroxyl group reach more favorable adsorption energies (up to −1.85 eV). This behavior suggests that the interaction is mainly controlled by charge redistribution from the rGO surface, and coordination effects. In contrast to the hydroxyl–functionalized systems, no water formation is observed, and the epoxy functional groups largely remain structurally intact after relaxation, with the exception of Figure 2C. Consequently, the interaction mechanism is dominated by electrostatic and coordination effects, without significant chemical reconstruction at the interface.

The present results further indicate that the stability of the rGO lattice depends on the type of functional group. Hydroxyl–functionalized rGO may undergo local chemical reconstruction through hydroxyl dissociation and occasional water formation, partially restoring the graphene lattice. In contrast, epoxy–functionalized rGO largely preserves its original structure after relaxation, exhibiting only limited local rearrangements. Consequently, hydroxyl/rGO promotes stronger interfacial reactivity, whereas epoxy/rGO better maintains the structural integrity of the graphene framework.

### 3.3. Computational Modeling of Elastic Properties in Dry Partially Decalcified Alkali Charge-Balanced C–A–S–H Nanocomposites and Untreated Systems

**Figure 3:**
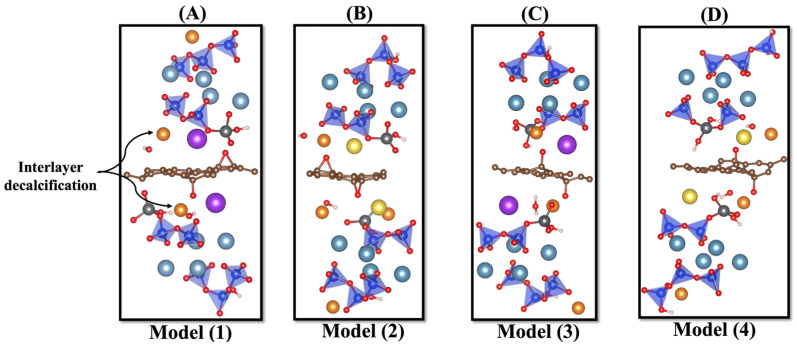
Fully relaxed structural models illustrating alkali–dry C–A–S–H nanocomposites with interlayer decalcification. Models (1–4) are based on Al–substituted C–S–H including interlayer Mg^2+^ and added interlayer hydroxyl groups, differing by charge–balancing cations and lattice type: Model (1): (**A**) interlayer K^+^ with epoxy/rGO lattice, Model (2): (**B**) interlayer Na^+^ with epoxy/rGO lattice, Model (3): (**C**) interlayer K^+^ with hydroxyl/rGO lattice, and Model (4): (**D**) interlayer Na^+^ with hydroxyl/rGO lattice. Color scheme applied throughout all figures: carbon atoms (C) are shown in brown, calcium cations (Ca^2+^) in greenish–turquoise, sodium cations (Na^+^) in yellow, potassium cations (K^+^) in purple, aluminum cations (Al^3+^) in dark gray within tetrahedra, silicon (Si) atoms in blue within tetrahedra, oxygen (O) atoms in red, and hydrogen (H^+^) in white.

**Figure 4:**
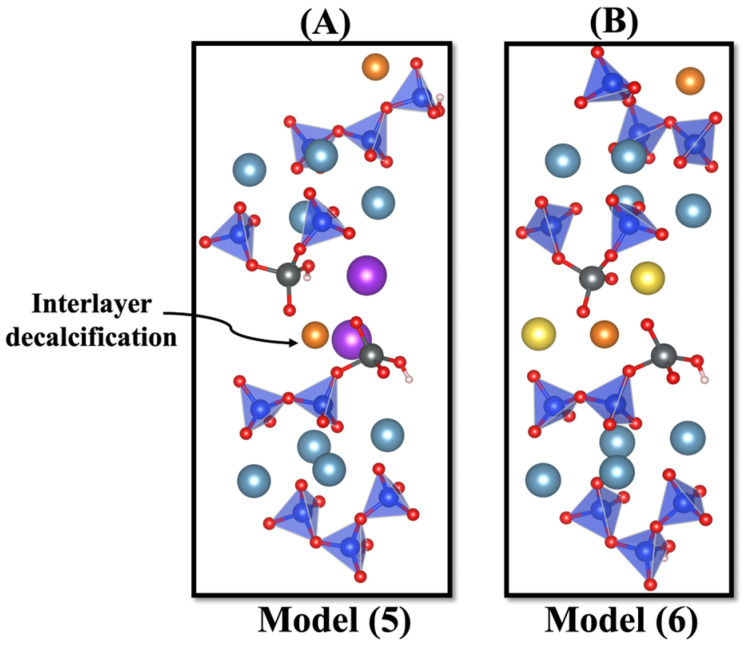
Fully relaxed structural models illustrating untreated alkali–dry C–A–S–H systems with interlayer decalcification. Models (5–6) are based on Al–substituted C–S–H including interlayer Mg^2+^, differing by charge–balancing cations: Model (5): (**A**) interlayer K^+^, Model (6): (**B**) interlayer Na^+^.

**Figure 5:**
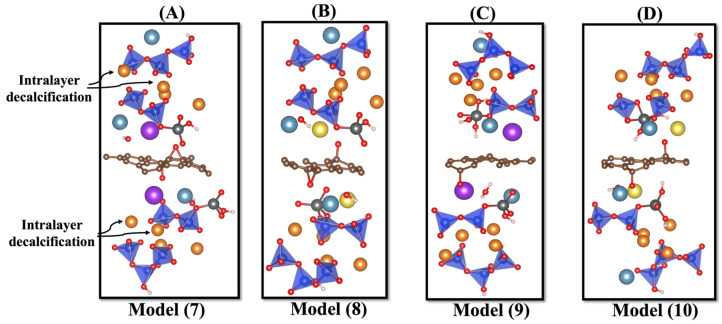
Fully relaxed structural models illustrating alkali–dry C–A–S–H nanocomposites with intralayer decalcification. Models (7–10) are based on Al–substituted C–S–H including intralayer Mg^2+^ and added interlayer hydroxyl groups, differing by charge–balancing cations and lattice type: Model (7): (**A**) interlayer K^+^ with epoxy/rGO lattice, Model (8): (**B**) interlayer Na^+^ with epoxy/rGO lattice, Model (9): (**C**) interlayer K^+^ with hydroxyl/rGO lattice, and Model (10): (**D**) interlayer Na^+^ with hydroxyl/rGO lattice.

**Figure 6:**
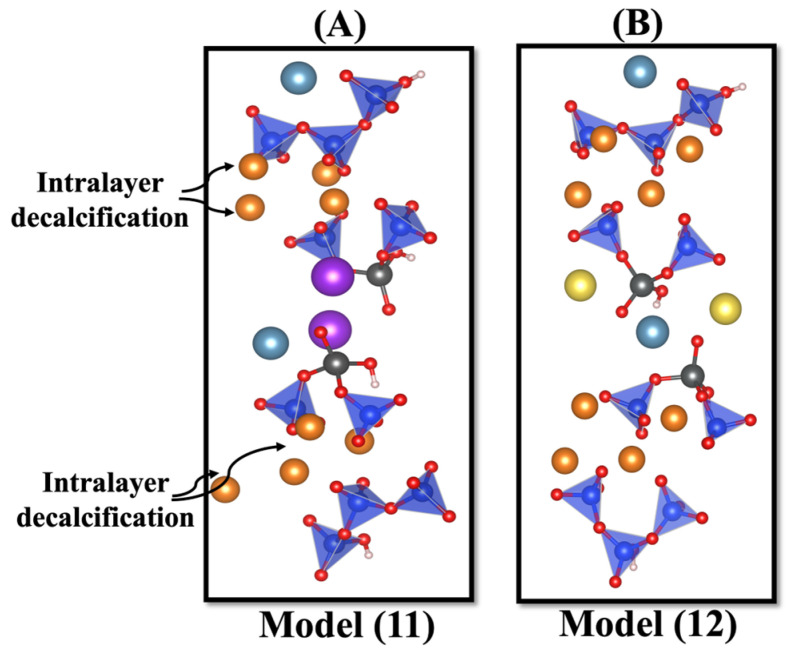
Fully relaxed structural models illustrating untreated alkali–dry C–A–S–H systems with intralayer decalcification. Models (11–12) are based on Al-substituted C–S–H including intralayer Mg^2+^, differing by charge-balancing cations: Model (11): (**A**) interlayer K^+^, Model (12): (**B**) interlayer Na^+^.

Figure 3, Figure 4, Figure 5 and Figure 6 present the fully relaxed atomistic structures of dry alkali charge-balanced C–A–S–H systems subjected to either interlayer or intralayer partial decalcification in the presence of Mg^2+^ substitution, with and without intercalated functionalized rGO lattices. The dry systems modeled, using a dehydrated tobermorite 14Å framework analogous to tobermorite 9Å, exhibit pronounced structural rearrangements following optimization, reflecting the substantial influence of decalcification and charge balancing on the local atomic environment. In all configurations, the removal of Ca^2+^ from either interlayer or intralayer positions induces local distortions in the silicate framework, which are partially compensated by Mg^2+^ substitution and alkali charge balancing through Na^+^ or K^+^ in the presence of Al^3+^. The presence of interlayer hydroxyl groups further contributes to electrostatic stabilization by compensating local charge imbalance associated with dangling oxygen atoms in silicate tetrahedra, thereby maintaining charge neutrality in the presence of Mg^2+^ or Ca^2+^ substitutions.

For the interlayer decalcified systems shown in Figure 3 and Figure 4, Mg^2+^ incorporation within the interlayer region results in a relatively compact structural arrangement after relaxation. This behavior can be attributed to the smaller ionic radius and higher charge density of Mg^2+^ compared with Ca^2+^, which promotes shorter metal–oxygen coordination distances and enhances local structural cohesion. The intercalation of functionalized rGO sheets further stabilizes the dry layered structure through interfacial interactions between oxygen-containing groups and C–A–S–H gel surface. In particular, Coulombic interactions between the polar epoxy/rGO or hydroxyl/rGO substrates and C–A–S–H surface moieties contribute significantly to interfacial stabilization, strengthening the adhesion between the rGO lattice and the silicate layers. Consequently, both epoxy/rGO and hydroxyl/rGO lattices remain closely associated with the silicate surface following structural optimization, indicating strong interfacial compatibility even under partially decalcified conditions. Compared with untreated models (Models 5–6), the nanocomposite structures (Models 1–4) exhibit visibly improved structural compactness, suggesting that rGO intercalation contributes to maintaining interlayer cohesion despite calcium depletion.

A different structural response is observed for intralayer decalcified systems (Figure 5 and Figure 6), where Mg^2+^ replaces Ca^2+^ directly within the silicate–bearing layer. Since intralayer Ca^2+^ are structurally integrated into the load–bearing framework of tobermorite, their removal induces stronger local distortions in the aluminosilicate skeleton compared with interlayer decalcification. Nevertheless, Mg^2+^ substitution partially restores local rigidity through stronger Mg–O bonding interactions, thereby limiting excessive framework destabilization. The presence of functionalized rGO in Models 7–10 similarly acts as a reinforcing phase; however, the resulting mechanical response appears more sensitive to the combined effects of interlayer charge-balancing cations and local intralayer decalcification.

**Figure 7:**
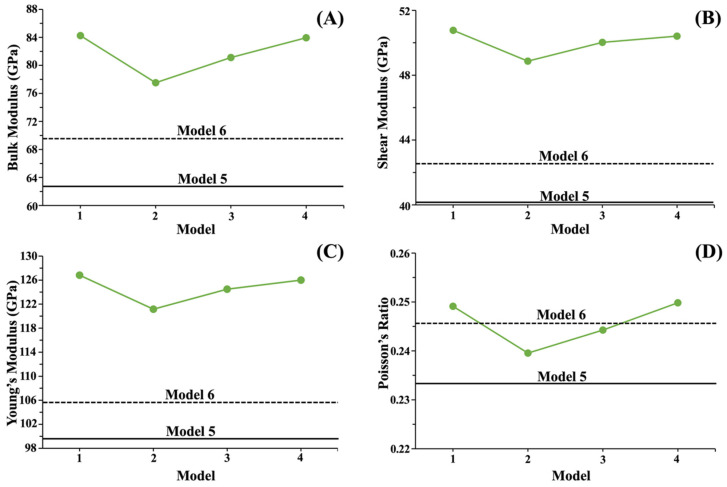
(**A**) bulk modulus, (**B**) shear modulus, (**C**) Young’s modulus, and (**D**) Poisson’s ratio for Models 1–6, with structural details provided in Table 1, are presented based on the data in Table 3. The purple lines denote the elastic properties of Models 1–4, representing alkali–dry C–A–S–H nanocomposites with interlayer decalcification. The solid (Model 5) and dashed (Model 6) black horizontal lines indicate the corresponding properties of alkali–dry C–A–S–H gel based on untreated tobermorite 9Å with interlayer decalcification, containing K^+^ and Na^+^, respectively, and serve as reference points to highlight the enhancement due to rGO intercalation.

**Figure 8:**
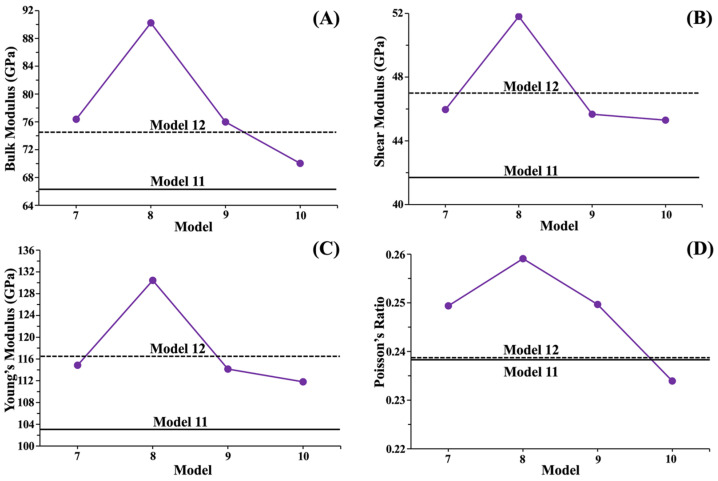
(**A**) bulk modulus, (**B**) shear modulus, (**C**) Young’s modulus, and (**D**) Poisson’s ratio for Models 7–12, with structural details provided in Table 2, are presented based on the data in Table 4. The purple lines denote the elastic properties of Models 7–10, representing alkali–dry C–A–S–H nanocomposites with intralayer decalcification. The solid (Model 11) and dashed (Model 12) black horizontal lines indicate the corresponding properties of alkali–dry C–A–S–H gel based on untreated tobermorite 9Å with intralayer decalcification containing K^+^ and Na^+^, respectively, and serve as reference points to highlight the enhancement due to rGO intercalation.

Table 1 and Table 2 summarize the structural details of the alkali–dry C–A–S–H models, including nanocomposite and untreated models with interlayer and intralayer decalcification, respectively. The models differ according to the charge–balancing cation (K^+^ or Na^+^), Mg^2+^ substitution, and the presence of epoxy/rGO or hydroxyl/rGO lattices. The computed elastic properties summarized in Table 3 and Table 4 and illustrated in Figure 7 and Figure 8, respectively, indicate that rGO intercalation substantially improves the mechanical performance of dry alkali charge–balanced C–A–S–H systems. This enhancement reflects a synergistic reinforcement mechanism involving (i) structural densification induced by Mg^2+^ substitution and (ii) interfacial stabilization (load transfer) enabled by rGO intercalation.

Representative C–S–H gel structures based on tobermorite 14Å have previously been investigated by Izadifar et al. [4], demonstrating that graphene-based reinforcement can improve structural stability and elastic properties through interfacial interactions. In addition, Santos Rego et al. [15] reported that Mg^2+^ substitution in pristine tobermorite 9Å and 11Å structures enhances stiffness and bulk modulus due to structural densification and shorter Mg–O bond lengths. Although the present models include alkali charge balancing, Al^3+^ substitution, partial decalcification, and rGO reinforcement, the observed improvements in elastic properties follows similar trends reported in these previous studies [4,15,18]. The relatively high elastic moduli obtained in the present work can be attributed to the crystalline nature of the atomistic models, together with the combined effects of Mg^2+^ substitution and rGO reinforcement. Figure 7 and Figure 8 illustrate the elastic properties of dry alkali charge–balanced C–A–S–H systems with interlayer and intralayer decalcification, respectively, based on the data presented in Table 3 and Table 4. In both cases, rGO intercalation significantly improves the mechanical performance relative to untreated models. For interlayer decalcification (Figure 7), the nanocomposite models (1–4) exhibit a more uniform enhancement, indicating that stiffness loss induced by calcium removal can be effectively compensated through Mg^2+^ substitution and interfacial reinforcement by functionalized rGO. Hydroxyl/rGO–containing systems generally exhibit slightly higher stiffness than epoxy/rGO counterparts, suggesting more effective stress transfer across the nanocomposite interface. Moreover, K^+^–containing systems tend to show marginally higher elastic moduli than Na^+^ analogs, likely due to improved electrostatic stabilization within the interlayer region.

In contrast, intralayer decalcification (Figure 8) exhibits a more sensitive mechanical response, reflecting the greater structural perturbation caused by calcium removal from the load–bearing silicate framework. Nevertheless, rGO reinforcement remains highly effective, with Model 8 (Na^+^ + epoxy/rGO) exhibiting the highest elastic properties among all dry systems, indicating a particularly favorable interaction between localized Na^+^ charge balancing and epoxy/rGO reinforcement. However, rGO intercalation does not universally improve elastic properties. Notably, Model 10 (Na^+^ + hydroxyl/rGO) exhibits lower elastic properties than untreated Model 12, indicating that hydroxyl/rGO reinforcement may become mechanically unfavorable under specific intralayer decalcification environments. Overall, interlayer decalcification results in more uniform mechanical enhancement, whereas intralayer systems show stronger sensitivity to local chemistry and reinforcement conditions.

### 3.4. Computational Modeling of Elastic Properties in Hydrated Partially Decalcified Alkali Charge-Balanced C–A–S–H Nanocomposites and Untreated Systems

**Figure 9:**
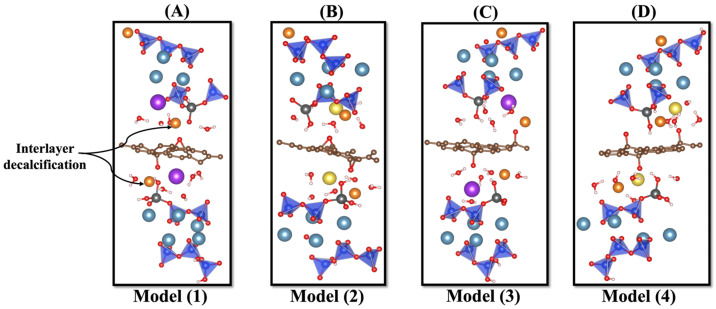
Fully relaxed structural models illustrating alkali–hydrated C–A–S–H nanocomposites with interlayer decalcification. Models (1–4) are based on Al-substituted C–S–H including interlayer Mg^2+^ and added interlayer hydroxyl groups, differing by charge–balancing cations and lattice type: Model (1): (**A**) interlayer K^+^ with epoxy/rGO lattice, Model (2): (**B**) interlayer Na^+^ with epoxy/rGO lattice, Model (3): (**C**) interlayer K^+^ with hydroxyl/rGO lattice, and Model (4): (**D**) interlayer Na^+^ with hydroxyl/rGO lattice.

**Figure 10:**
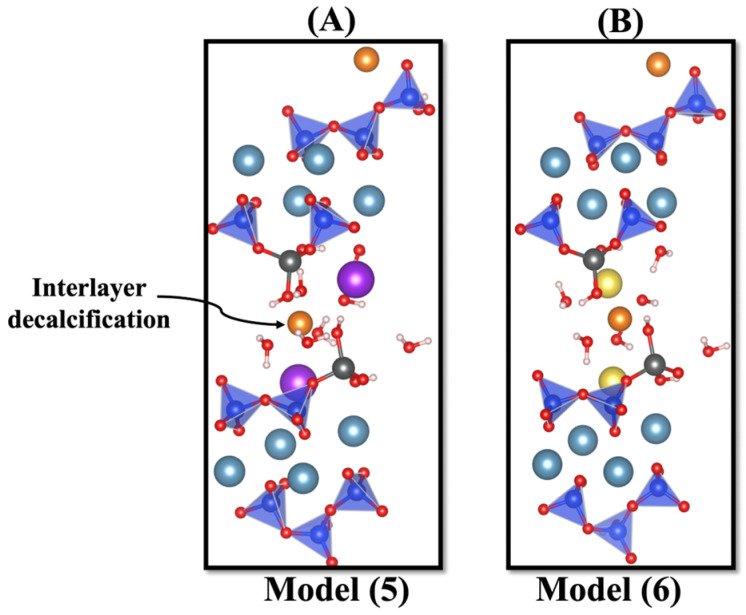
Fully relaxed structural models illustrating untreated alkali–hydrated C–A–S–H systems with interlayer decalcification. Models (1–2) are based on Al–substituted C–S–H including interlayer Mg^2+^, differing by charge–balancing cations: Model (1): (**A**) interlayer K^+^, Model (2): (**B**) interlayer Na^+^.

**Figure 11:**
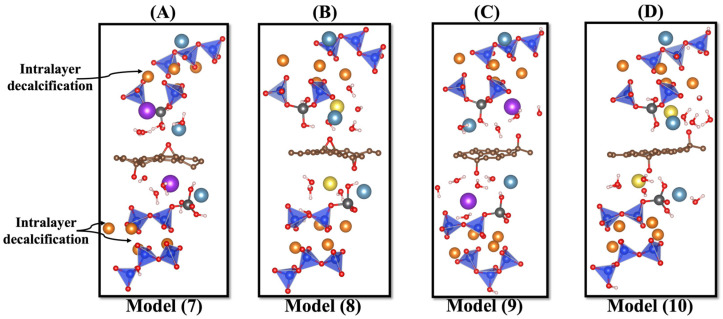
Fully relaxed structural models illustrating alkali–hydrated C–A–S–H nanocomposite with intralayer decalcification. Models (7–10) are based on Al–substituted C–S–H including intralayer Mg^2+^ and added interlayer hydroxyl groups, differing by charge–balancing cations and lattice type: Model (7): (**A**) interlayer K^+^ with epoxy/rGO lattice, Model (8): (**B**) interlayer Na^+^ with epoxy/rGO lattice, Model (9): (**C**) interlayer K^+^ with hydroxyl/rGO lattice, and Model (10): (**D**) interlayer Na^+^ with hydroxyl/rGO lattice.

**Figure 12:**
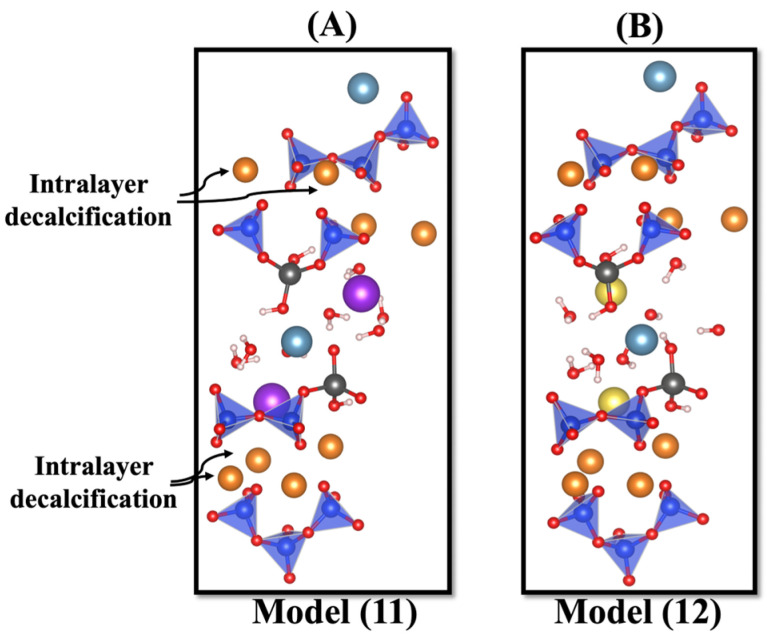
Fully relaxed structural models illustrating untreated alkali–hydrated C–A–S–H systems with intralayer decalcification. Models (1–2) are based on Al–substituted C–S–H including intralayer Mg^2+^, differing by charge–balancing cations: Model (1): (**A**) interlayer K^+^, Model (2): (**B**) interlayer Na^+^.

Hydrated alkali charge-balanced C–A–S–H systems subjected to partial interlayer and intralayer decalcification are presented in Figure 9, Figure 10, Figure 11 and Figure 12. Compared with dry systems, the incorporation of interlayer water molecules leads to a more stabilized and structurally cohesive framework, reflecting the important role of hydration in maintaining interlayer integrity. In all hydrated configurations, water molecules contribute to local structural stabilization through hydrogen bonding. As a result, the hydrated systems generally exhibit reduced structural perturbation following decalcification compared with their dry counterparts.

For interlayer decalcification (Figure 9 and Figure 10), Mg^2+^ incorporation together with alkali charge balancing preserves interlayer cohesion despite calcium depletion. The presence of epoxy/rGO and hydroxyl/rGO further promotes structural stability through interfacial interactions with C–A–S–H gel surface. In contrast to dry systems, hydration appears to reduce differences between epoxy– and hydroxyl–functionalized lattices, indicating that interlayer water contributes to more uniform interfacial stabilization across different rGO functionalization. Likewise, the hydrated nanocomposite systems (Models 1–4, Figure 9) exhibit improved structural cohesion relative to untreated systems (Models 5–6, Figure 10), indicating that rGO reinforcement remains effective under hydrated conditions.

A comparable structural trend is observed for intralayer decalcification (Figure 11 and Figure 12), with hydrated systems maintaining overall structural stability following optimization. Intralayer calcium removal from these sites induces greater local structural perturbation than interlayer decalcification. Nevertheless, interlayer hydration partially mitigates the local structural distortions induced by intralayer calcium removal, leading to more stable relaxed structures than in dry systems.

**Figure 13:**
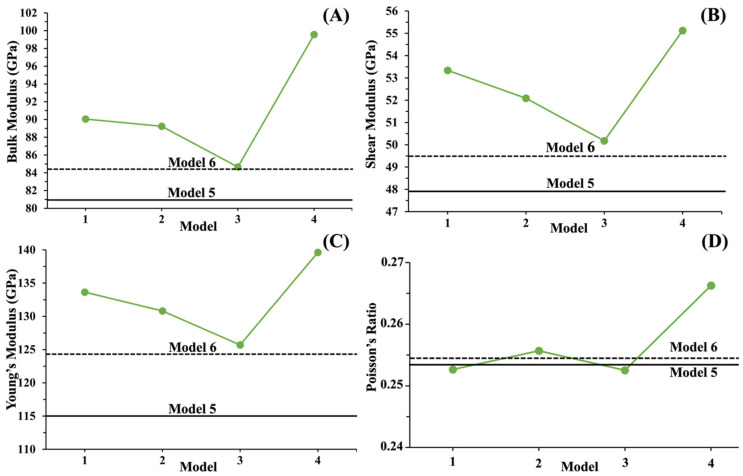
(**A**) bulk modulus, (**B**) shear modulus, (**C**) Young’s modulus, and (**D**) Poisson’s ratio for Models 1–6, with structural details provided in Table 5, are presented based on the data in Table 7. The purple lines denote the elastic properties of Models 1–4, representing alkali–hydrated C–A–S–H nanocomposites with interlayer decalcification. The solid (Model 5) and dashed (Model 6) black horizontal lines indicate the corresponding properties of alkali–hydrated C–A–S–H gel based on untreated tobermorite 14Å with interlayer decalcification containing K^+^ and Na^+^, respectively, and serve as reference points to highlight the enhancement due to rGO intercalation.

**Figure 14:**
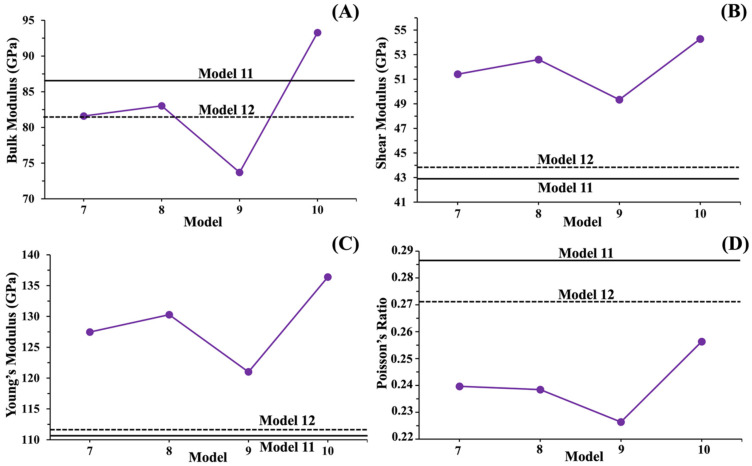
(**A**) bulk modulus, (**B**) shear modulus, (**C**) Young’s modulus, and (**D**) Poisson’s ratio for Models 7–12, with structural details provided in Table 6, are presented based on the data in Table 8. The purple lines denote the elastic properties of Models 7–10, representing alkali–hydrated C–A–S–H nanocomposites with intralayer decalcification. The solid (Model 11) and dashed (Model 12) black horizontal lines indicate the corresponding properties of alkali–hydrated C–A–S–H gel based on untreated tobermorite 14Å with intralayer decalcification containing K^+^ and Na^+^, respectively, and serve as reference points to highlight the enhancement due to rGO intercalation.

Table 5 and Table 6 summarize the structural details of alkali–hydrated C–A–S–H models, including nanocomposite and untreated models with interlayer and intralayer decalcification, respectively. The models differ according to the charge–balancing cation (K^+^ or Na^+^), Mg^2+^ substitution, and the presence of epoxy/rGO or hydroxyl/rGO lattices. The corresponding elastic properties are summarized in Table 7 and Table 8 and illustrated in Figure 13 and Figure 14, respectively.

Figure 13 and Figure 14 demonstrate that hydration generally enhances the elastic performance of alkali charge–balanced C–A–S–H systems. A largely identical trend in elastic properties is observed for both interlayer and intralayer decalcifications under hydrated conditions (Figure 13 and Figure 14). For interlayer decalcification (Figure 13), most nanocomposite models exhibit improved elastic properties relative to untreated models, indicating that rGO intercalation generally contributes to mechanical reinforcement under hydrated conditions. Among the hydrated interlayer systems, Model 4 (Na^+^ + hydroxyl/rGO) exhibits the highest elastic properties of the nanocomposite models, while untreated systems (Models 5–6) show comparatively lower elastic properties. Overall, the elastic properties of hydrated interlayer systems remain relatively consistent across different rGO functionalizations and charge–balancing cations, indicating effective mechanical stabilization under hydrated conditions. For intralayer decalcification (Figure 14), hydration relatively improves the effectiveness of rGO reinforcement. Notably, Model 10 (Na^+^ + hydroxyl/rGO), which exhibited mechanically unfavorable behavior in the dry state under intralayer decalcification, shows a pronounced enhancement in elastic properties under hydrated conditions, surpassing untreated Model 12. This transition highlights the important stabilizing role of interlayer water, which likely promotes stronger interfacial coupling and reduces structural stress concentrations near decalcified intralayer regions. Nevertheless, Model 10 exhibits the highest Young’s modulus (136.38 GPa) and bulk modulus (93.28 GPa) among hydrated intralayer systems, indicating a particularly favorable synergistic interaction between Na^+^ charge balancing, hydroxyl/rGO functionalization, and hydration.

### 3.5. Discussions

**Figure 15:**
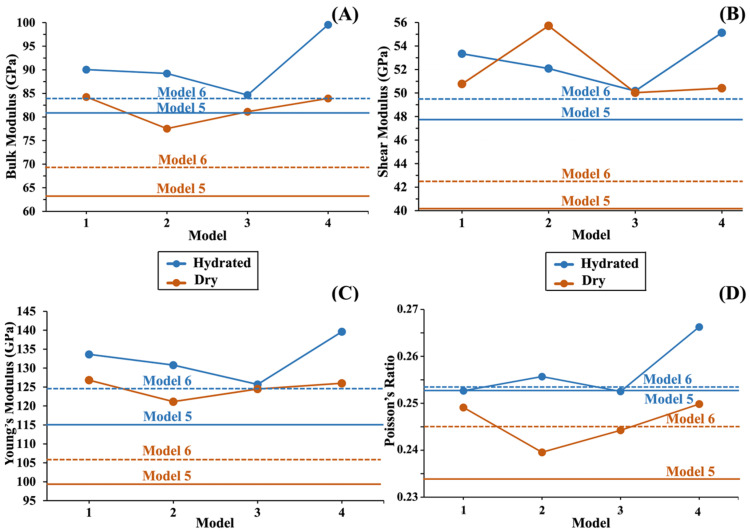
Comparison of (**A**) bulk modulus, (**B**) shear modulus, (**C**) Young’s modulus, and (**D**) Poisson’s ratio for dry and hydrated states of models 1–6, as shown in Figure 7 and Figure 13.

The comparison between dry and hydrated systems shown in Figure 15 reveals that interlayer hydration plays an important role in improving the elastic performance of partially decalcified alkali charge–balanced C–A–S–H nanocomposites. Overall, the presence of interlayer water increases elastic moduli, indicating enhanced structural stabilization of partially decalcified interlayer frameworks. For interlayer decalcification (Figure 15A), the alkali–hydrated C–A–S–H systems containing interlayer water exhibit an increase in bulk modulus of approximately 4–19% in nanocomposite systems and 21–29% in untreated systems, indicating improved resistance to volumetric deformation. Likewise, for interlayer decalcification (Figure 15B), Young’s modulus increases by approximately 1–11% in nanocomposite systems and 16–17% in untreated systems. The greatest improvement is observed for Model 4 (Na^+^ + hydroxyl/rGO), where Young’s modulus increases from 126.00 to 139.61 GPa (≈10.8%), accompanied by a bulk modulus increase of ≈18.6%. These findings suggest that interlayer water contributes to improved structural stability, enhanced interfacial cohesion, and partial mitigation of the mechanical perturbation induced by calcium depletion.

**Figure 16:**
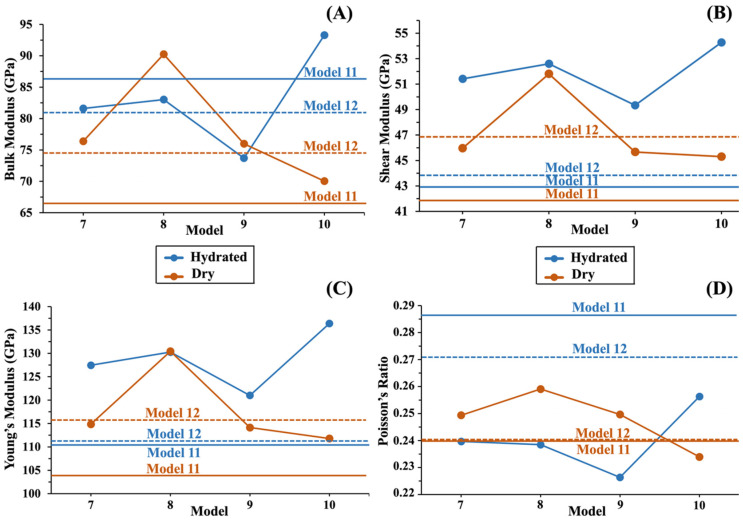
Comparison of (**A**) bulk modulus, (**B**) shear modulus, (**C**) Young’s modulus, and (**D**) Poisson’s ratio for dry and hydrated states of Models 7–12, as shown in Figure 8 and Figure 14.

A similar but more pronounced trend is observed for intralayer decalcification (Figure 16), where the presence of interlayer water substantially improves the elastic properties of systems that exhibited mechanically unfavorable behavior in the dry state. Most notably, Model 10 (Na^+^ + hydroxyl/rGO), which exhibited reduced elastic properties in the dry state, undergoes a marked improvement upon hydration, with Young’s, shear, and bulk modulus increasing by approximately 22.0%, 19.8%, and 33.2%, respectively. In contrast, Model 8 (Na^+^ + epoxy/rGO) and Model 9 (K^+^ + hydroxyl/rGO) exhibit slight reductions in bulk modulus of ≈8.0% and ≈3.0%, respectively, accompanied by marginal changes in Young’s modulus (≈−0.1% and ≈+6.0%, respectively), indicating that hydration-induced reinforcement is not universally favorable but remains strongly dependent on local chemistry and interfacial configuration. In contrast, Model 7 (K^+^ + epoxy/rGO) and Model 10 (Na^+^ + hydroxyl/rGO) exhibit concurrent improvements in both Young’s and bulk moduli, with Young’s modulus increasing by ≈11.0% and ≈22.0%, respectively, accompanied by bulk modulus enhancements of ≈6.8% and ≈33.2%. This consistent increase in both elastic indicators suggests a stronger hydration-induced stabilization response under intralayer decalcification. This pronounced recovery suggests that interlayer water becomes particularly important when calcium depletion occurs within the load-bearing silicate framework, where local structural perturbations are inherently more severe. Overall, intralayer hydration not only improves the elastic properties of alkali charge-balanced C–A–S–H systems but also reduces the mechanical sensitivity associated with local decalcification environments and rGO functionalization.

Compared with untreated hydrated systems (Models 5–6), rGO–treated nanocomposites (Models 1–4) generally exhibit higher elastic properties, with Young’s modulus increasing by approximately 5–16% and bulk modulus by ≈4–18%, depending on charge-balancing cation and rGO functionalization. A comparable but more model-dependent trend is observed for hydrated intralayer decalcification (Figure 16). Relative to untreated systems (Models 11–12), rGO–treated nanocomposites (Models 7–10) generally exhibit higher Young’s modulus, with improvements ranging from ≈9 to 22%, indicating enhanced stiffness despite intralayer calcium depletion. In contrast, bulk modulus exhibits a more variable response, ranging from a decrease of ≈15% to an increase of ≈15%, reflecting stronger sensitivity to local chemistry and interfacial configuration under intralayer decalcification. Most notably, Model 10 (Na^+^ + hydroxyl/rGO) exhibits the strongest enhancement, with Young’s and bulk moduli increasing by ≈22.3% and ≈14.6%, respectively, relative to untreated Model 12.

A comparable trend has also been reported by Izadifar et al. [4] in C–S–H nanocomposites based on tobermorite 14Å reinforced with epoxy/rGO and hydroxyl/rGO lattices, where the presence of interstitial water generally contributed to improved elastic properties, although the extent of enhancement depended on the local interfacial configuration and charge–compensation environment. In particular, the incorporation of interstitial water was found to favor higher elastic moduli in most cases, supporting the present observation that hydration can partially mitigate structural perturbations associated with calcium depletion and improve the mechanical response of partially decalcified C–A–S–H systems. Collectively, these findings indicate that the mechanical response of partially decalcified alkali charge–balanced C–A–S–H systems is governed not only by Mg^2+^ substitution and rGO reinforcement, but also by the synergistic role of interlayer water in stabilizing local atomic environments and mitigating decalcification–induced mechanical degradation. Experimental studies have shown that Mg^2+^ incorporation can contribute to the stabilization of silicate hydrate structures, influence the formation of Mg–containing binding phases in cementitious materials, and improve the long–term assessment of the geochemical evolution of cement–clay interfaces in geological repository environments, highlighting the importance of considering Al–containing M–S–H phases [31,35,61,62]. Similarly, graphene–based additives have been reported to improve the mechanical performance of cementitious composites through reinforcement and improved interfacial bonding. The atomistic results obtained in the present study provide a mechanistic explanation for these experimental observations by showing that Mg^2+^ substitution promotes local structural densification, while functionalized rGO enhances interfacial stabilization within partially decalcified C–A–S–H structures. These combined effects lead to improved elastic properties and support the potential of Mg–modified graphene-reinforced binders for the development of durable low–carbon cementitious materials.

Although stronger adsorption energies indicate enhanced interfacial stabilization between functionalized rGO and the C–A–S–H matrix, a direct quantitative correlation with the elastic properties cannot be established. The elastic response is additionally influenced by Mg^2+^ substitution, alkali charge balancing, hydration state, local structural relaxation, and the degree of decalcification. Nevertheless, the favorable adsorption energies observed for the nanocomposite systems suggest improved interfacial load transfer and structural cohesion, which contribute to the enhanced elastic properties. Furthermore, the effect of hydration depends on the local structural environment of each model. Interlayer water promotes structural stabilization through hydrogen bonding, electrostatic screening, and improved ion coordination. Consequently, hydration produces a greater enhancement in elastic properties for structures with larger distortions or charge imbalance, whereas more stable dry models exhibit a smaller response.

In future work, these partially decalcified systems will be employed to explore thermodynamically favorable carbonation reactions across various surface orientations, surface–catalyzed CO_2_ adsorption, and calcium carbonate formation pathways in alkali charge–balanced C–A–S–H phases, with the broader objective of improving mineralization–based carbon capture strategies in sustainable cementitious materials.

## 4. Conclusions

This study employed density functional theory (DFT) calculations to investigate the combined effects of interlayer and intralayer partial decalcification, Mg^2+^ substitution, and epoxy–and hydroxyl–functionalized reduced graphene oxide (rGO) reinforcement on the structural stability and elastic properties of alkali charge–balanced C–A–S–H systems under dry and hydrated conditions. The main findings are summarized as follows:

1. Functionalized rGO together with Mg^2+^ substitution generally enhances the mechanical performance of partially decalcified C–A–S–H through structural densification and interfacial stabilization.
2. Under dry conditions, interlayer decalcification exhibited more uniform mechanical enhancement, whereas intralayer decalcification showed greater sensitivity to local chemistry and reinforcement conditions.
3. Hydration through the presence of interlayer water further improved the elastic performance in most systems, increasing Young’s modulus by approximately 1–11% and bulk modulus by 4–19% for interlayer decalcified nanocomposites, respectively.
4. Intralayer decalcified systems exhibited stronger but more model–dependent responses, with improvements reaching up to ≈22% in Young’s modulus and ≈33% in bulk modulus.
5. The hydrated Na^+^ + hydroxyl/rGO configuration demonstrated substantial mechanical recovery under intralayer decalcification, highlighting the important synergistic role of hydration, alkali charge balancing, and rGO functionalization.
6. Overall, the combined incorporation of Mg^2+^ and functionalized rGO emerges as a promising strategy to mitigate decalcification–induced mechanical degradation in alkali charge–balanced C–A–S–H systems, providing atomistic guidance for the design of durable low–carbon cementitious nanocomposites.

## Figures and Tables

**Table 1:** Structural details of six alkali–dry C–A–S–H models, including both nanocomposite and untreated models, incorporating interlayer decalcification.

Model	K^+^	Na^+^	Inter-Mg	Epoxy/rGO	Hydroxyl/rGO	Dry C–A–S–H
**1**	Included	Excluded	Included	Included	Excluded	Included
**2**	Excluded	Included	Included	Included	Excluded	Included
**3**	Included	Excluded	Included	Excluded	Included	Included
**4**	Excluded	Included	Included	Excluded	Included	Included
**5**	Included	Excluded	Included	Excluded	Excluded	Included
**6**	Excluded	Included	Included	Excluded	Excluded	Included

**Table 2:** Structural details of six alkali–dry C–A–S–H models, including both nanocomposite and untreated models, incorporating intralayer decalcification.

Model	K^+^	Na^+^	Intra-Mg	Epoxy/rGO	Hydroxyl/rGO	Dry C–A–S–H
**7**	Included	Excluded	Included	Included	Excluded	Included
**8**	Excluded	Included	Included	Included	Excluded	Included
**9**	Included	Excluded	Included	Excluded	Included	Included
**10**	Excluded	Included	Included	Excluded	Included	Included
**11**	Included	Excluded	Included	Excluded	Excluded	Included
**12**	Excluded	Included	Included	Excluded	Excluded	Included

**Table 3:** The Young’s modulus, shear modulus, bulk modulus, and Poisson’s ratio of Models (1–6) described in Table 1.

Model	1	2	3	4	5	6
**Young’s Modulus (GPa)**	126.82	121.15	124.49	126.00	99.33	105.89
**Shear Modulus (GPa)**	50.76	48.87	50.03	50.41	40.20	42.51
**Bulk Modulus (GPa)**	84.25	77.53	81.13	83.95	62.92	69.73
**Poisson’s Ratio**	0.249	0.239	0.244	0.250	0.233	0.245

**Table 4:** The Young’s modulus, shear modulus, bulk modulus, and Poisson’s ratio of Models (7–12) described in Table 2.

Model	7	8	9	10	11	12
**Young’s Modulus (GPa)**	114.85	130.45	114.14	111.80	103.85	116.27
**Shear Modulus (GPa)**	45.96	51.80	45.66	45.30	41.91	46.89
**Bulk Modulus (GPa)**	76.37	90.24	75.99	70.03	66.38	74.50
**Poisson’s Ratio**	0.249	0.259	0.250	0.234	0.239	0.239

**Table 5:** Structural details of six alkali–hydrated C–A–S–H models, including both nanocomposite and untreated systems, incorporating interlayer decalcification.

Model	K^+^	Na^+^	Inter-Mg	Epoxy/rGO	Hydroxyl/rGO	Hydrated C–A–S–H
**1**	Included	Excluded	Included	Included	Excluded	Included
**2**	Excluded	Included	Included	Included	Excluded	Included
**3**	Included	Excluded	Included	Excluded	Included	Included
**4**	Excluded	Included	Included	Excluded	Included	Included
**5**	Included	Excluded	Included	Excluded	Excluded	Included
**6**	Excluded	Included	Included	Excluded	Excluded	Included

**Table 6:** Structural details of six alkali–hydrated C–A–S–H models, including both nanocomposite and untreated systems, incorporating intralayer decalcification.

Model	K^+^	Na^+^	Intra-Mg	Epoxy/rGO	Hydroxyl/rGO	Hydrated C–A–S–H
**7**	Included	Excluded	Included	Included	Excluded	Included
**8**	Excluded	Included	Included	Included	Excluded	Included
**9**	Included	Excluded	Included	Excluded	Included	Included
**10**	Excluded	Included	Included	Excluded	Included	Included
**11**	Included	Excluded	Included	Excluded	Excluded	Included
**12**	Excluded	Included	Included	Excluded	Excluded	Included

**Table 7:** The Young’s modulus, shear modulus, bulk modulus, and Poisson’s ratio of Models (1–6) described in Table 5.

Model	1	2	3	4	5	6
**Young’s Modulus (GPa)**	133.64	130.81	125.69	139.61	115.01	124.16
**Shear Modulus (GPa)**	53.34	52.09	50.18	55.13	47.88	49.50
**Bulk Modulus (GPa)**	90.05	89.24	84.64	99.56	80.98	84.30
**Poisson’s Ratio**	0.253	0.256	0.253	0.266	0.253	0.254

**Table 8:** The Young’s modulus, shear modulus, bulk modulus, and Poisson’s ratio of Models (7–12) described in Table 6.

Model	7	8	9	10	11	12
**Young’s Modulus (GPa)**	127.47	130.28	121.01	136.38	110.48	111.54
**Shear Modulus (GPa)**	51.41	52.60	49.34	54.28	42.93	43.86
**Bulk Modulus (GPa)**	81.60	83.02	73.70	93.28	86.37	81.43
**Poisson’s Ratio**	0.2396	0.2385	0.2264	0.2563	0.2867	0.2717

## Data Availability

The original contributions presented in this study are included in the article. Further inquiries can be directed to the corresponding author.
